# Perception of Hong Kong Teenagers and Young Adults on Esports Participation: A Qualitative Study Using Theory of Planned Behavior

**DOI:** 10.3389/fpsyg.2021.650000

**Published:** 2021-07-09

**Authors:** Ming Yu Claudia Wong, Pak-Kwong Chung, Kailing Ou, Ka-Man Leung

**Affiliations:** ^1^Department of Sport, Physical Education and Health, Hong Kong Baptist University, Kowloon, Hong Kong; ^2^Department of Health and Physical Education, Education University of Hong Kong, Tai Po, Hong Kong

**Keywords:** perception, attitude, teenager and young adult, Hong Kong, theory of planned behavior, eSports

## Abstract

Esports is a rapidly growing industry worldwide, and it is making significant inroads in Hong Kong as well. However, owing to debates regarding the distinction between Esports and video gaming and the potential negative effects of engaging in Esports, its development in Hong Kong is still in its infancy. Therefore, this qualitative study investigated the perceptions and attitudes of teenagers and young adults toward Esports development and engagement, using the theory of planned behavior. Twenty-five teenagers and young adults (male = 24, female =1) participated in this study, with their ages ranging from 15 to 29 years. Our results revealed the views of Hong Kong teenagers and young adults on the beneficial and deleterious outcomes (goal setting and achievement, physical health, socialization and teamwork, psychological benefits, academics and time distribution, physical strain, negative social image, and perception toward sport participation), subjective norms (parents, peers, teachers, and modeling effect), and barriers and facilitators (balance between academics and Esports, capability, career prospects and future reality, psychological benefits, and peer encouragement and support) of participating in Esports. Additionally, the results of this qualitative study may contribute toward a deeper understanding of Hong Kong Esports players to develop a theory of planned behavior construct for capturing the beliefs and perceptions of Hong Kong teenagers toward Esports.

## Introduction

Esports is a rapidly growing industry worldwide. Owing to this rapid global development of Esports, the Hong Kong Special Administrative Region (HKSAR) government has decided to follow in the footsteps of the global community and promote Esports as “a new sector with economic development potential” (Hong Kong Policy Address 2017/18). The HKSAR government has also announced implementations regarding the maintenance of the local gaming industry and the development of a new gaming technology in its policy address, including a total of 100 million Hong Kong dollar investment to promote Esports, such as providing venues, technological development, and nurturing talent. Despite there were measures taken by the Hong Kong government to support the development of the Esports industry, there is still a long way to go compared to other Asian countries and regions such as Korea and the Mainland where with massive Esports industry scale (Cybersport, 2018).

Currently, the sports industry is divided over the definition of Esports. According to the Oxford Dictionary (2018), Esport refers to “a multiplayer video game played competitively for spectators, typically by professional gamers.” Esports exhibit characteristics similar to traditional sports in that participants require skills, training, tactics, rules and regulations, teamwork, and prizes. In fact, the International Olympic Committee (IOC) has agreed that Esports “can be considered a sporting activity” and “can provide a platform for engagement with the Olympic Movement” (Reuters, 2017). Esports was also provided a trial run in the PyeongChang 2018 Winter Olympic Games before the opening ceremony. Furthermore, the Olympic Council of Asia has already included Esports in two Asian Games (i.e., 2018 and 2022) as a demonstration and an official competition event. However, *Esports* is still hardly distinguishable from *video gaming* for the general population. Hence, research studies have expressed significant concern about the mental and physical health problems potentially caused by intensive participation in Esports, or in other words, video gaming. Psychological and physical issues prevalent among teenagers include gaming addiction, decreased sleeping time, reduced physical activity, attention problems, screen dependency disorder, and other health issues (e.g., Rehbein et al., 2010; Wang et al., 2014). These negative health impacts have tarnished the social image of Esports, leading to difficulties in developing Esports and different perceptions and attitudes toward its development in Hong Kong. The Hong Kong Federation of Youth Groups (HKFYG) (2018) conducted a study regarding the development of Esports in Hong Kong, and about 1,400 adolescents aged 15–29 years were included in this study. Results indicated that 38.3% of the respondents had watched and 13.4% participated in Esports competitions, respectively, in the 6-month period prior to the survey. More than 60% of the respondents supported the development of Esports by the Hong Kong government. Additionally, the FinTech community of Hong Kong, based on studies of Hong Kong Esports development, underscored the importance of Esports to be equipped with a positive public image for it to become a mainstream sport (Office of the Government Chief Information Officer, 2018). However, these studies were neither completely data-driven nor did they possess a theoretical basis compelling enough to illustrate the perceptions and attitudes of Hong Kong teenagers toward Esports development and engagement. The benefits of using theory in qualitative health studies are that the questions can be asked in a flexible way and thus enables the data to be revealed in a more sophisticated approach. In addition, the usage of theory could have helped to define the analysis strategy including questions about the level of analysis and how analytical decisions are described (Kelly, 2010).

Furthermore, according to the structuring of the Esports Research Agenda (Cranmer et al., 2021), it has mentioned that the different cultures of different regions could have led to diverse Esports environments. The agenda has also mentioned that how to scale up the development of the industry in Esports developing regions and organize smaller live leagues should be focused in future research. Given that the Esports industry is well-established in the Asian market like China, Taiwan, and Korea, whereas, Hong Kong is also situated in this cultural context notwithstanding, Hong Kong's Esports environment is still developing. Therefore, to investigate students' perceptions of Esports participation in Hong Kong are central to this study in which to be in line with the research gap mentioned by the Esports Research Agenda. Additionally, understanding the current state of Esports development in Hong Kong can serve as a reference for those countries and regions where Esports is still in a developing stage.

The purpose of this study was thus, to investigate the participation of teenagers and young adults in Esports using the theory of planned behavior (TPB; Ajzen, 1991, Figure 1). TPB postulates that behaviors and behavioral intentions are shaped by an individual's attitude (i.e., the attribute of a particular behavior), subject norms (i.e., whether a significant person would approve or disapprove of the behavior), and perceived behavioral controls (i.e., anticipated obstacles that may inhibit behavior). Other than these direct predictors of behavior and behavioral intentions, the above three determinants have their own indirect measures, called salient beliefs, namely behavioral, normative, and control beliefs (Ajzen, 2006). Behavioral beliefs are operationalized as the summed product of the likelihood of potential outcomes weighted by the values of the outcomes, such as the beneficial and deleterious effects of participating in Esports. Normative beliefs include how significant the referent affecting the behavior is and the motivation to comply with the referent, such as parents' approval and public's perception of playing Esports. Control belief is the sum of the strength of control belief and the perceived power of control belief to facilitate and inhibit behavior, such as lack of skills or knowledge required to play Esports. This theory is content-free and has been highly utilized in predicting physical activity behavior (Sun et al., 2015; Zhang et al., 2019) and problematic online video gaming behavior (Alzahrani et al., 2017).

**Figure 1:**
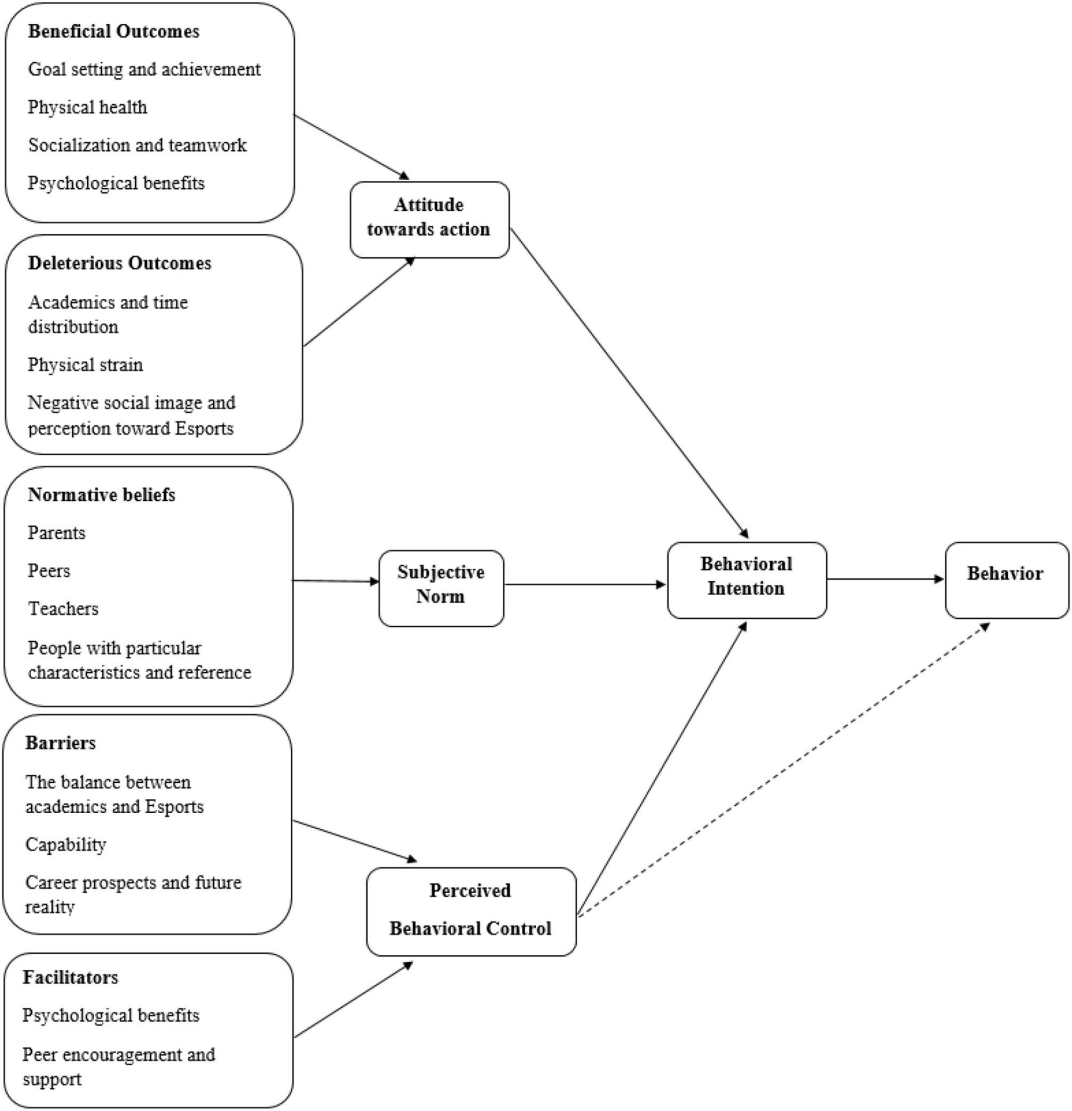
Theory of planned behaviors of Esports participation (Ajzen, 1991).

## Methodology

### Participants

Twenty-five teenagers and young adults participated in this study, which met the qualitative sample size suggested by Glaser et al. (1968). Teenagers and young adults were defined as individuals studying in secondary schools or universities (aged 15–29 years) in Hong Kong. This age range was chosen with reference to previous studies conducted by the Office of the Government Chief Information Officer (2018) and the Hong Kong Federation of Youth Groups (2018), where most of the population playing Esports was aged between 15 and 29 years (studying in secondary schools or universities). According to the guidelines suggested by Francis et al. (2004), participants were stratified considering (1) whether they were secondary or university students and (2) whether they had participated in an Esports competition in the last 6 months. Among the 25 participants (24 males), 52% were university students, and the mean age was 18.72 years (range = 15–26 years). Forty percent of participants had participated in Esports competitions in the last 6 months. Regarding gaming type, Table 1 shows the most popular game among participants was League of Legends (LOL) (64%), followed by PlayerUnknown's Battlegrounds (PUBG mobile) (44%).

**Table 1:** Demography of participants.

**No**.	**Age**	**Gender**	**Years of playing Esports**	**Participated in Esports competitions in the last 12 months**	**Gaming type**	**Education**
1	20	Male	5 years	Yes	PUBG	University
2	19	Male	3 years	Yes	PUBG	University
3	15	Male	5 months	Yes	PUBG	Secondary school
4	16	Male	4 years	Yes	PUBG	Secondary school
5	15	Male	4.5 years	Yes	PUBG	Secondary school
6	20	Male	N/A	Yes	LOL	University
7	22	Male	N/A	Yes	LOL	University
8	18	Male	N/A	Yes	LOL	Secondary school
9	20	Male	N/A	Yes	LOL	University
10	20	Male	5.5 years	Yes	LOL	University
11	24	Male	10 years	Yes	LOL	University
12	21	Male	8 years	Yes	LOL	University
13	21	Female	N/A	Yes	LOL	University
14	20	Male	8 years	Yes	LOL	University
15	20	Male	Beginning	Yes	LOL	University
16	16	Male	5 years	Yes	LOL; PUBG; Minecraft	Secondary school
17	16	Male	7 years	Yes	Minecraft; PUBG	Secondary school
18	26	Male	12 years	Yes	Decathlon	University
19	23	Male	16 years	Yes	PUBG	University
20	16	Male	10 years	Yes	PUBG	Secondary school
21	18	Male	10 years	Yes	PUBG; LOL	Secondary school
22	16	Male	5.5 years	Yes	LOL; Rainbow Six	Secondary school
23	16	Male	5 years	Yes	LOL	Secondary school
24	15	Male	6 years	Yes	LOL	Secondary school
25	16	Male	4 years	Yes	LOL; Rainbow Six	Secondary school

### Method

Focus group interviews (five in a group) were conducted to investigate the participation of teenagers and young adults in Esports using purposive sampling. Focus groups have been used in numerous fields to explore perceptions of concepts, products, or ideas. Open questions accompanied by interactive settings enable participants to talk freely with each other, which stimulates their ideas (Leung and Savithiri, 2009). Current advanced technology enables a focus group to be conducted without any geographical restrictions, either online or face-to-face (Stewart and Shamdasani, 2017). The interview guide was developed according to the guidelines suggested by Fishbein and Ajzen (2011) based on the TPB construct.

### Procedure

This study was conducted between February and March 2020. All research activities were reviewed and approved by the Hong Kong Baptist University Research Ethics Committee (HAS97-1810828). An invitation letter and a cover letter detailing the aims and procedures of our study were presented to our external partner (i.e., ER Esports Limited), who provides Esports services in Hong Kong. With the agreement of the manager of our partnered organization, information regarding possible interview times, dates, and locations of the five focus groups was disseminated through social media platforms, such as the WhatsApp group. Interested participants were enrolled by contacting the manager, and their interview times and venues were confirmed. Due to COVID-19, both face-to-face and online focus group interviews were optional for participants. Two onsite groups of interviews were conducted in a meeting room of our partner company, and the remaining three were conducted via a Zoom meeting. Before the focus group interview, participants were informed about the objectives of the study, their right to leave the project anytime, and that their personal data would be kept confidential and destroyed after the completion of this research. Interviewees who had participated in online interviews were requested to move to a quiet place with access to a stable-speed network before the interview began. Two investigators (KLO, MYW) experienced in moderating focus group discussions and familiar with the purpose of the study were involved. They were female, knowledgeable about focus groups and Esports, and had no prior relationships with the interviewees. All the interviews were audiotaped. During the interviews, some observational notes were also recorded by the investigators to enhance the accuracy of the transcripts and data analysis. Participants had the right to view the transcripts. After the completion of the focus group interviews, participants were given an HKD100 supermarket voucher for their contribution to our study. The interviews took an average of 65.2 min (range = 42–94 min) to complete.

## Data Analysis

All the interviews were transcribed verbatim and checked against the file notes. A combination of transcripts and observational notes provides the researcher with a deep understanding of the experiences and processes under study (Persson et al., 2014). The transcripts were qualitatively analyzed through thematic content analysis using the software NVIVO 12, and analytic induction was used to describe the perceptions of the participants (Elo and Kyngäs, 2008). Two investigators (MYW and KLO) analyzed the interview contents independently by creating tags as provisional names. Two investigators then combined the tags with similar meanings and labeled the sub-categories, categories, and themes following the TPB constructs (behavioral, normative, and control beliefs). Afterwards, the coded transcripts were grouped into the latest codebook and compared and discussed by the investigators again (Graneheim and Lundman, 2004). During the coding process, discussion among coders can facilitate the development of thematic codes to achieve quality data. Principal investigators (CPK and LKM) read all the codes, reflected and confirmed the basis among the coders, and checked inter-coder reliability. Interviewees were invited to do checking relating to the accuracy of the data such as interview scripts and the results analyzed. As described by Miles and Huberman (1994), analysis of qualitative data involves data reduction, data display, and drawing and verifying conclusions. In order to classify the characteristics of the participants, quotations were labeled by a short key; for example, G1M1 indicated the first male participant from group one. Furthermore, data collection and data analysis were done interactively in order to refine and enrich the empirical data (Glaser et al., 1968; Corbin and Strauss, 2014). Data analysis has been engaged during the process of data gathering, for revision of the interview guide and for further data collection.

## Results

### Perceived Behavioral Beliefs

Perceived behavioral beliefs refer to the potential outcomes of participating in Esports that can be beneficial or deleterious. It has been accorded an important role in determining the attitudes toward Esports participation among the participants of this study.

#### Beneficial Outcomes

##### Goal Setting and Achievement

Interviewees believed that clear goal setting and strong belief in goal achievement were positive outcomes of participating in Esports.

One of the male participants (G1M2) stated: “*when you are interested in one thing, you have a goal in your life … some high school students don't know what they want to do in the future, but you have already planned your goals … and you will never regret it (Interviewer: Can I take it that you reflect more on yourself when you participate in Esports and training?) Sure*.

Two male interviewees (G2M3 and G4M1) reported: “*the advantage of participating in Esports is that I can achieve my dream*” and “*when I am playing videogames, my purpose is to win the game … I want to achieve my goal*.”

One female interviewee (G3M4) stated: “*Esports seem like another future way out (as a kind of achievement) for those who have poor grades*”.

##### Physical Health

Long-term highly intensive Esports training requires participants to be physically strong, and some of them felt their physical health had improved after playing Esports.

Two male participants (G1M4 and G1M3) reported: “*I think Esports is good for the physical and mental (health) because we all have so much fun when we played Esports (Interviewer: I'm so glad you said that. I know you need a lot of training to play Esports, but is it true that training can help your physical health?) definitely, because I keep stretching every day when I have to concentrate on playing a game, so doing physical activities does help “(G1M3)” I feel the same way, I was an undisciplined person before, but I go on a diet, my physical health is better after I started playing Esports, Because I will push myself to do better in all aspects.”*

“*Playing Esports can strengthen my physical health, train brain reactions and eyes-hands-coordination*.”(G3M1).

##### Socialization and Teamwork

Participants also described that playing Esports could improve their socialization, such as making friends, teamwork, and communication skills.

Two male participants (G1M2 and G4M4) reported: “*when you see your classmates playing games, you may join them because you have something in common with them … so you can make lots of friends*” and “*There is no jet lag in the Esports world … I had made lots of foreigner friends, some of them really came to Hong Kong and we met in real life*.”

“*Our minds are in sync … we discussed and reflected on ourselves after training.”* (G2M5)

“*At the beginning, I was not good at communicating with others. However, after playing Esports, my teamwork and communication skills improved*.”(G4M5).

##### Psychological Benefits

Apart from improving their physical health, some of the interviewees believed that playing Esports could also strengthen their psychological health. For example, judgment, concentration, self-affirmation, stress reduction, and perseverance improved for the participants.

Regarding judgment and concentration, one male participant (G4M4) reported: “*Esports training can speed up my judgment*” and G1M4 stated: “*we take it (Esports) very seriously … as we may review our performance in the last match that can foster our sense of concentration … we all focus on it (Esports).”*

Concerning self-affirmation and stress reduction, one male participant (G1M5) stated: “*I think I can reach the highest level through self-enforcement … I won the championship last year which is a meaningful thing, it made me feel self-affirmed.”* One participant (G2M1) reported: “*a benefit of playing Esports is that it reduces stress*.”

About perseverance, two male participants (G1M3 and G1M4) stated: “*I won't give up even if I met a higher-ranked opponent,” “I always wonder how to reach a higher rank*.”

#### Deleterious Outcomes

While there were several benefits of Esports, respondents also mentioned some of the obstacles they faced while playing Esports. Most of the barriers they perceived concerned academics, time management, and physical strain.

##### Academics and Time Distribution

Esports training might take up most of the time; however, several schools require students to concentrate on their studies, as Esports is not a mainstream sport in Hong Kong. One participant (G4M3) complained: “*It (Esports) is a high-risk low return sport, when you spend lots of time training, your studies or other developments will be affected, this is the disadvantage*.” Another participant (G1M5) also stated: “*The allocation of time is challenging after a long training period because sometimes you play too late, and you will have no energy in the next early morning class*.”

Two male interviewees exclaimed: (G2M1) “*the biggest problem of Esports is you spend 24 h on it … it's very challenging to balance study and Esports … my schedule is so full and I struggle*,” *(G2M5)* “*Other than time consuming, It takes lots of time and energy, you might spend 10–14 h sitting there*.”

##### Physical Strain

Although participants mentioned that their physical health had improved, some felt that they had physically hurt themselves while playing Esports. Two male participants (G1M2 and G2M2) stated: “*Long screening time causes diminution of vision … and spinal curvature*” and “*Not everyone's computer settings meet the health standards … it might cause cervical spondylosis*.”

Two male participants (G3M5 and G4M4) reported: “*one of the Esports champions in Hong Kong named Lok Wai Kin, his hand was shaking after the game … another professional athlete also suffers from chronic wrist problems*” and “*I began to find that my wrist was easily inflamed*.”

##### Negative Social Image and Perception Toward Esports Participation

When enquiring about what else came to their minds when they participated in Esports, participants stated that they were concerned about the perception of the public, such as their peers and the audience.

One male participant (G1M1) reported: “*my classmate doubts if there is a differentiation between Esports and merely playing computer games, they don't understand why I waste my time on this*.”

“…*sometimes I would be affected by the eyes of the audience … this is an important psychological factor, you need to be bold and show off in public … this would be pressurizing because lots of people watch how you play*.” (G4M3).

### Normative Beliefs

Normative beliefs express the social pressure and social norms that interfere with Esports participation of participants; this interference typically comes from individuals or groups who would approve or disapprove of their participation. These individuals or groups could be significant people around them or people possessing specific characteristics that could be idolized.

#### Parents

Reviewing the transcripts revealed that most parents disapproved of their children participating in Esports. However, participants stated that the attitudes of their parents changed when they earned achievements in an Esports game.

One male participant (G4M1) said that “*I think the first people who are against it are of course parents because the thinking of every parent is traditional, they hope we can get good grades, get a good job, earn money, and buy a house. They think that playing Esports is a waste of time*.” Another participant (G4M2) agreed: “*cannot agree more, their stereotype of playing Esports is that it affects studies seriously*.”

One male participant (G2M5) reported: “*at the beginning, when I started playing Esports, no one supported me, including my parents, they crushed my confidence*.”

However, the perception of their parents changed once participants began earning achievements in Esports games. One male participant (G1M3) stated: “*they disapproved at first until they saw me on TVB, then they realized that Esports is a new industry, so they accepted it gradually*.” Another participant (G5M6) reported: “*my mom was very much against me until she saw that I won the competition, then she began to tolerate my hobby*.” G5M2 agreed: “*she was against me too, but she changed her mind after watching my interview … she hasn't fully supported me yet but she has stopped arguing with me, she stays in a wait-and-see mode*.”

Two male participants (G2M2 and G5M4) said: “*they support me all the time, they encourage me to try something different”* and “*they support me (interviewer: do they have any other comment when they saw you playing Esports?) it should not affect my studies, for example*.”

#### Peers

Most interviewees received support from their friends or teammates, and their success in Esports has earned them the admiration of their friends. Participant G1M2 stated: “*all my friends are supporting me*” and G1M3 reported: “*all my friends have been playing Esports for more than 10 years … they support me*.”

“*At first I was an outcast in the class, my classmates made fun of my Esports level, however, after I won more competitions, they came to me and started consulting me regarding the skills needed for playing Esports*.”(G2M3)

“*They didn't support me at first, but when I got results, they all came to learn from me*.”(G2M4).

#### Teachers

Many interviewees described that their teacher held positive attitudes toward Esports and encouraged them to try new things. One participant (G1M5) stated: “*my teammates and I were troublemakers in class … until 1 day, our teacher saw us focusing on Esports which was unbelievable, so she started to support us*.”

Two participants (G2M3 and G4M5) said: “*my professor asked me to chase my dream, to try it (Esports) if I get a chance*” and “*my teacher asked us to participate in competitions … the younger teachers are more supportive of our diversified development*.”

However, one participant (G3M3) stated that his teacher stayed firm in his negative attitude toward Esports, he said: “*The career life span of Esports is too short*.”

Noteworthy, most of the participants who mentioned about the views and attitudes of teachers were secondary school students. It was expected that secondary school students tended to prioritize their studies, while perceived their teachers as a more significant social groups that have a negative impact on their participation in Esports, compared to university participants.

#### People With Particular Characteristics and Reference

When discussing what kind of people or characteristics may affect their engagement in Esports after a chaotic period, interviewees listed those who were optimistic, had financial support, and were persistent. “*I knew someone who engaged in Esports for so many years, but they didn't believe they would earn achievements or reach a high level, they gave up finally*.” (*G*1M3); “*The supported technical equipment of Esports is expensive and in great demand*.” (G4M3); “*I have a friend who played in an Esports team with a meager income, but his perseverance and hard work pushed him to be a professional player, now his income is abundant*.” (G1M5). When participants were struggling to persist in playing Esports, most of them would inspire themselves by the experiences of Esports athletes. “*I am 22 years old now, which means my age is relatively high in Esports. However, my idol, Tom, won the world Esport Championship when he was 27 years old, so I have no excuse to quit*.” (G1M4).

### Perceived Behavioral Control

Perceived behavioral control is a belief about factors that may facilitate or impede participants' performance in Esports.

#### Barriers

##### The Balance Between Academics and Esports

As indicated in the demographic information shown in Table 1, all of the participants were students. Therefore, academic study was the principal and one of the major issues that they mentioned. Many participants revealed that they faced difficulties balancing academics and playing Esports. Also, there tended to be conflicts between participants and their parents over spending considerable time playing Esports, which parents thought of as playing “video games,” and spending less time and effort on their studies. The issue of balancing academics and playing Esports indicated a time management problem for the participants.

A participant (G4M2) stated that “*Esports will affect academics because playing Esports not only requires time for training but also the time spent exploring and investigating the setting of a new game, how to play with new skills and techniques, as well as how to cooperate with teammates*.”

As a result, participants encountered difficulties in managing time between their studies and playing Esports. However, they also expressed that they could play Esports only while they were still students. This is because “*the lifespan of an Esports player is too short, you can only play till you are approximately 26 years old, and the most competitive age is between 15 and 20 years*” (G2M3). Therefore, they had to sacrifice numerous other activities in their life to participate in Esports.

Due to the competition edge among the Esports field, Esport players tended to be younger, secondary school participants are more concerned about their studies than university participants, thus it is more difficult for them to balance their time and energy between their studies and Esports. This illustration has also revealed a self-contradictory situation within the Esport players.

##### Capability

As mentioned above (G2M3), the lifespan of a professional Esports player is rather short due to changes in the capabilities of a person as they age (Thompson et al., 2014). These capabilities include physical capability as well as Esports skills and ability. Both Esports participants and non-Esports participants agreed that capability is one of the motivations to keep them going.

First, participants thought that with the increase in age, their physical capability, especially their reaction time and sightseeing spot accuracy, would reduce; in other words, they would react slower to the game and competition compared to the younger players. This was how one of the participants described the change in their physical capability:

“*It is all due to your own physical body. When you get older, you may not be able to react to the game as fast as you could before. Your eyesight will be poorer and may reduce your competitiveness. Therefore, if your competitors are younger than you, their reactions will be faster, and you would need to boost your energy immensely in order to remain competitive*.”*(G2M2)*

Other than that, gaming skills and ability also influenced their views and beliefs in being part of Esports. This was because the participants thought that

“*Current Esports playing or game playing is not the same as playing the older games, which only require sufficient equipment and physical energy, without any specific skills. However, nowadays, Esports, which is called Sports, involves fairness. The competition involves not only what normal sports do, which is purely based on physical fitness and energy, but Esports also require skills and continuous improvement in order to adapt to a new game version. Training alone is not enough because a game will have different versions from time to time, with different characters and functions, such as guns or other weapons; therefore, if you are not sensitive enough to the skills and improving your performance skills, your competitiveness and ability will weaken and affect your future development*.”*(G4M2)*.

Therefore, participants believed that gaming skills and ability were very important, and any regression and lack of improvement in all forms of their capability would induce them to discontinue playing Esports.

##### Career Prospects and Future Reality

The career prospects of playing Esports professionally were indicated as too narrow in Hong Kong. The reality of Hong Kong's Esports development did not provide sufficient opportunities for Esports players to compete and perform to their ability for Esports to become a serious career prospect for them. In addition, an Esports athlete could hardly secure any other occupation, either part-time or after retiring from being a professional Esports athlete. Hence, this led to another issue of a lack of income and monetary support for living expenses.

A participant mentioned that “*Money is the second big issue, being a professional Esports athlete is being really poor in Hong Kong (Interviewer:I can understand that when you get to a higher level you find that Hong Kong doesn't have the resources to support you, so you have choose to be a part-time player?*). *But there is no part-time players in Esports area.” (G5M1)*

The participant indicated that the income of a full-time Esports player in Hong Kong could hardly support the daily living expenses of the player. This is because the Esports competition system in Hong Kong is not mature enough to attract sponsors and support a big amount of winning monetary prize. Moreover, the electronic equipment for professional Esports players is costly as well. Therefore, the unstable and low income of Esports players has affected their belief and intention to continue participating in professional Esports. Whilst these illustrations and perceptions were mainly stated by the university participants, who tended to be concerned more about their career, either in Esports or their future career prospect. It is because university students had passed the golden age of being Esports players and tended to face more livelihood problems than that of secondary school students, so they are concerning more about the future. However, the unstable income of being Esports professional players in Hong Kong is a deterrent to them.

#### Facilitators

##### Psychological Benefits

Despite all the above-mentioned barriers, there were two main facilitators that could motivate participants to engage in Esports. The first facilitator was the psychological benefits of participating in Esports competitions. Among all the psychological benefits mentioned by the participants, the most common was the feeling of accomplishment after competing in an Esports competition and obtaining satisfactory results.

A participant expressed his accomplishment as, “*You have been working so hard to beat the other competitors, and finally reach the ranking that you wanted. You will feel so happy, and realize that the time you spent before was worthwhile*.” *(G4M1)*

In addition to experiencing a feeling of accomplishment, participants believed that playing Esports could show their achievements and prove their abilities to others, hence increasing their self-confidence, especially when they used self-designed playing skills or techniques to win a game.

##### Peer Encouragement and Support

Another major facilitator was encouragement and support from peers, mainly friends and teammates. According to the responses of the participants, most of their friends played video games or Esports. Their teammates were mostly friends they knew before they began playing, and who had then formed a team to participate in Esports. Therefore, support from friends and teammates tended to be a motivator for them; they could also share their skills, techniques, and thoughts on the games with each other, further forming a social bond with each other. Yet, some participants also revealed differences in motivation derived from teammates and friends.

A participant (G2M1) expressed that the support from teammates stems from “*tacit understanding and cooperation*,” he thought that “*Having a group of good teammates to support you and keep you company is very difficult (interviewer: sounds like looking for a marriage partner haha), haha even more difficult than finding a person to get married with*, for example, if you were 0.5 s slower in a competition, you have already lost a lot.” Another participant (G4M1) also mentioned that “*Esports is a team-based sport, a team with one heart, goal, or direction …, could fully support each other in a competition and achieve good results together*.”

Therefore, the unity of and support within a team could be strong facilitators for Esports players, preventing them from giving up playing Esports.

Besides, friends who were not teammates could act as a different kind of motivator. One of the participants (G3M2) expressed that “*Friends are one of the facilitators because some of my friends were at a rank higher than me. So, I think that we could work harder together to improve and reach a higher rank*.”

As a result, despite both teammates and friends having shown different forms of motivation toward interviewees' participation in Esports, peer support was shown to be a facilitator of Esports participation. Figure 1 summarizes the intention of Esports participation using TPB framework.

## Discussion

Esports is a booming industry in Hong Kong. However, the development of Esports is still considered to be in its infancy. There are various debatable and controversial factors present within the potential Esports players, community, society, and consequent influence to the development of teenagers and young adults, especially the potential Esports players' intentions or reactions to participating in Esports. This qualitative study is designed to investigate the participation of Hong Kong teenagers in Esports, using the theory of planned behavior (TPB; Ajzen, 1991). Our results revealed Hong Kong teenagers and young adults' views on the beneficial and deleterious outcomes (goal setting and achievement, physical health, socialization and teamwork, psychological benefits, academics and time distribution, physical strain, and negative social image and perception toward Esports participation), subjective norms (parents, peers, teachers, and people with particular characteristics and reference) as well as barriers and facilitators (balance between academics and Esports, capability, career prospects and future reality, psychological benefits, and peer encouragement and support) of participating in Esports.

### Perceived Behavioral Beliefs and Control

Perceived behavioral beliefs and control factors have been combined for discussion since their interrelationship was revealed during the analysis of our results. Participants indicated the advantages and disadvantages of participating in Esports, yet they were shown to be the facilitators and barriers to their participation as well as participation intention.

The most significant advantages, in other words, the facilitators of participating in Esports could be indicated as the psychosocial benefits of playing Esports. Participating in Esports was described as enabling self-affirmation, reducing stress, fulfilling accomplishments while achieving goals, and being trained to be perseverant. In addition, the social network built upon playing Esports allows them to form social commitment and bonding, in which socialization generates participants' sense of belonging to the Esports team and supporting each other. A previous study of professional Esports players also indicated that there was an increase in their level of personal happiness, mainly developed through achieving better self-esteem and stronger commitment in the Esports field (Guo et al., 2020). Similarly, another qualitative study investigating positive personal development through Esports demonstrated that the major benefits of playing Esports were a sense of commitment and initiative to achieve personal standards and goals, thus gaining self-improvement (Johnston et al., 2013; Carbonie et al., 2018) as well as a sense of achievement and success when showcasing one's competence in Esports (Schwartz, 1992; Carbonie et al., 2018). In addition, offering and receiving feedback between teammates and coaches also allows players to enhance their communication skills (Johnston et al., 2013; Carbonie et al., 2018). As such, Hong Kong teenagers and young adults in this study reported their views and attitudes regarding the advantages and facilitators of participating in Esports akin to those expressed in the current literature.

On the other hand, the disadvantages of Esports were also indicated as barriers to Hong Kong teenagers and young adults participating in Esports. First, time management and balance between academics and Esports are a common concern among participants and even traditional sports athletes. A qualitative study on the Hong Kong water sports athletes' perception toward sports career engagement also showed similar results: academic achievements were shown to be the main responsibility of teenagers; hence, difficulties in managing academic engagements would lead to a lower intention to engage in sports (Wong, 2017). Second, similar results were also applied to the poor social perception toward Esports and its narrow career development. In a study by Wong (2017), it was noted that Esports is plagued by a poor social image. Social image refers to images that individuals enforce on certain behaviors or the type of people who performs those behaviors (Burrow, 1924). The nature of Esports had been criticized by society until it was clarified by the International Olympic Committee (Reuters, 2017). Hence, Esports participants were misunderstood as playing video games, which is seen as an addictive behavior. Moreover, the career development of professional athletes, either playing traditional sports or Esports, was shown to be restricted by age, career prospects, academic qualifications, and physical capability. Therefore, these kinds of social images might have affected people's perceptions of Esports participation, including players, parents, peers, teachers, or even the general public, thus, further inhibiting the development of Esports in Hong Kong. Finally, the physical condition and capability of Esports players were shown to be one of the disadvantages and barriers to Esports participation. All sports athletes, including Esports players, might suffer from sports injuries and lowered physical competence with an increase in age. Although research has found that participating in Esports could improve a person's intellectual ability, and Esports players were also shown to engage in physical exercise training within their Esports training routine (Kari and Karhulahti, 2016; Freeman and Wohn, 2017), the participants of this study reported that they had suffered from injuries, such as joint strain and long-term eye fatigue, due to the prolonged usage of mouse and keyboards. As such, under the strain of such long training hours and the increase in age, the skills and physical capabilities of an Esports player might be viewed as less competitive compared to the younger generation of players. Beyond the barrier of physical incapability, the short athlete lifespan of Esports players has also minimized the career development of Esports players and influences Hong Kong teenagers' participation in Esports.

### Normative Beliefs

Normative beliefs refer to the subjective norms that the beliefs of an individual will be affected by social pressure from their significant others, mainly parents and peers.

Social norms and images have been seen to affect parents' and teachers' views and attitudes toward certain behaviors, for instance, engaging in the sports industry (Jonasson and Thiborg, 2010; Wong, 2017; Jiow et al., 2018). In Esports, parents have also expressed their negative perceptions and attitudes toward their children playing Esports, whereas surprisingly, teachers tend to show greater acceptance of Esports participation of their students. This may be because teachers trained under the modern form of education tend to adopt the learner-centered approach, in which teachers are encouraged to support students' personal growth and all-rounded development. This approach could further enhance students cognitively, behaviorally, and affectively by allowing them to participate in activities that they feel more motivated to, instead of just focusing on traditional knowledge and skills (Boulton-Lewis et al., 2001; Shek et al., 2017).

Other than parents and teachers, certain specific characteristics of some people or social groups, such as sports idols or professional Esports players, also interfere with teenagers' beliefs about participating in Esports. These characteristics tend to be intangible, such as capability, positive mentality, and perseverance, which could determine who is treated as a role model. Although normative beliefs are defined as social beliefs acting as external factors influencing teenagers and young adults' participation, participants in the interviews expressed a desire to equip themselves with these characteristics, and that could be an intrinsic motivation for them to continue to participate in Esports in the foreseeable future. A sense of empowerment and competence to achieve satisfaction are shown to cause individuals to be more confident in internalizing the regulations of participating in a particular behavior and facilitate internal motivation, thus, leading them to feel less (Davidson and Garrido, 2015).

### Limitations and Strengths

It should be noted that only one female participant could be contacted, thus showing a gender imbalance in the Esports field. Our study may fail to uncover the potential factors that influence female Esports players. Yet, the gender imbalance has indicated that Esports development among Hong Kong girls may be much narrower than among the boys (Wang et al., 2014; Su et al., 2019). Notwithstanding, the recruited participants were stratified according to their education level as well as their Esports competition experience. The current study involved both secondary school and university students who had participated in interschool or public Esports competitions in the past 6 months, thus ables comparisons between the stratification. This stratification has allowed the inclusion of more comprehensive sources of data and enhanced the level of trustworthiness of the study (Pitney, 2004).

Besides, to the best of our knowledge, this study is the foremost theoretical-based qualitative Esports study on Hong Kong teenagers and Esports players. TBP has been a prominent theory in predicting human behavior across a plethora of contexts, ranging from health-related behaviors (Godin and Kok, 1996; Armitage and Conner, 1999), volunteer behavior (Okun and Sloane, 2002; Greenslade and White, 2005), physical activity and exercise (Hausenblas et al., 1997; Potwarka, 2015), to sport consumption behaviors (Cunningham and Kwon, 2003; Kaplanidou and Gibson, 2010; Eddosary et al., 2015), yet not to Esports participation intentions and behaviors. In line with this view, the TPB model looks pertinent in the current research context, especially when eSports is at the beginning of its development in Hong Kong. The perceived influencing variables, including attitude, subjective norms, perceived behavioral control, negative factors, positive factors, facilitators, barriers and the related Esports development, can be considered as obstacles or factors that affect Hong Kong teenagers' behavior intention toward eSports as well.

Given that the Esports industry is considered as an ongoing trend within the globe and nonetheless within Hong Kong, Hong Kong teenagers would have substantially engaging in the field of Esports. The current research has then successfully provided the potential stakeholders of Esports, including parents, schools, Esports practitioners, Electronic companies and the HKSAR Government with a deeper investigation of Esports participants using the qualitative approach, which could also initiate an understanding of the disparity and interspaces among Esports players, social norms, and the Esports industry in Hong Kong. For instance, it provides the schools and teachers with a justification of students' excessive use of screen; or even provide the Hong Kong government or Hong Kong Esports Association with an overview of Hong Kong's current Esports environment, in which facilitate their future implementation and establishment of Esports regulations and better infrastructure; also to provide Esports practitioners or game companies with a deeper understanding toward developing Hong Kong's Esports markets.

## Conclusion

To conclude, this qualitative study has critically demonstrated the views of Hong Kong teenagers and young adults on the beneficial and deleterious outcomes and subjective norms of participating in Esports, as well as the barriers and facilitators that interfere with their participation. The interview results also indicated the development of Esports was in its infancy in Hong Kong, affecting its social perception and image and teenagers' Esports participation. Besides, as the current study was Part one of a Hong Kong public policy research project on “The development of Esports in Hong Kong,” the current qualitative study was useful to further a deeper understanding of Hong Kong Esports players to develop a TPB construct measure for capturing Hong Kong teenagers' beliefs and perceptions toward Esports. Moreover, in the later part of the research, the developed TPB construct measure for Esports will be utilized to examine Hong Kong Teenagers' Esports intention and behavior quantitatively. Also, a further investigation of Hong Kong Esports development as well as exploring the views and attitudes of the general public will be conducted in the later part of the research. Furthermore, future interventions could be developed for Esports players to improve the negative health consequences caused by long time training, for example, healthy screen time monitory, improving physical strain (chronic wrist and back) problems, study behaviors, nutrition, posture, and psychological measures.

## Data Availability Statement

The datasets presented in this article are not readily available because all qualitative data were restricted to public unless with participants authorization. Requests to access the datasets should be directed to 18481795@life.hkbu.edu.hk.

## Ethics Statement

The studies involving human participants were reviewed and approved by Hong Kong Baptist University Research Ethics Committee. Written informed consent to participate in this study was provided by the participants' legal guardian/next of kin.

## Author Contributions

P-KC and K-ML: conceptualization, methodology and validation. P-KC, K-ML, MW, and KO: review and editing. MW and KO: data collection, formal analysis, data curation, and writing–original draft preparation. All authors contributed to the article and approved the submitted version.

## Conflict of Interest

The authors declare that the research was conducted in the absence of any commercial or financial relationships that could be construed as a potential conflict of interest.

## Abbreviations

HKSAR: the Hong Kong Special Administrative Region
IOC: the International Olympic Committee
HKFYG: the Hong Kong Federation of Youth Groups
TPB: the Theory of Planned Behavior
LOL: League of Legends
PUBG mobile: PlayerUnkown's Battlegrounds.
